# Respiratory problems in children with esophageal atresia and tracheoesophageal fistula

**DOI:** 10.1186/s13052-017-0396-2

**Published:** 2017-09-05

**Authors:** Federica Porcaro, Laura Valfré, Lelia Rotondi Aufiero, Luigi Dall’Oglio, Paola De Angelis, Alberto Villani, Pietro Bagolan, Sergio Bottero, Renato Cutrera

**Affiliations:** 1 Respiratory Unit, Academic Department of Pediatrics, Bambino Gesù Children’s Hospital, IRCCS, Rome, Italy; 2 Department of Medical and Surgical Neonatology, Neonatal Surgery Unit, Bambino Gesù Children’s Hospital, IRCCS, Rome, Italy; 3 General Pediatrics and Pediatric Infectious Diseases Unit, Academic Department of Pediatrics, Bambino Gesù Children’s Hospital, IRCCS, Rome, Italy; 4 Digestive Surgery and Endoscopy Unit, Surgical Department, Bambino Gesù Children’s Hospital, IRCCS, Rome, Italy; 5 Laryngotracheal team, Airway Surgery Unit, Bambino Gesù Children’s Hospital, IRCCS, Rome, Italy

**Keywords:** Congenital malformations, Esophageal atresia, Tracheoesophageal fistula, Respiratory symptoms, Flexible bronchoscopy

## Abstract

**Background:**

Children with congenital esophageal atresia (EA) and tracheoesophageal fistula (TEF) have chronic respiratory symptoms including recurrent pneumonia, wheezing and persistent cough. The aim of this study is to describe the clinical findings of a large group of children with EA and TEF surgically corrected and the instrumental investigation to which they have undergone in order to better understand the patient’s needs and harmonize the care.

**Methods:**

A retrospective data collection was performed on 105 children with EA and TEF followed at Department of Pediatric Medicine of Bambino Gesù Children’s Hospital (Rome, Italy) between 2010 and 2015.

**Results:**

69/105 (66%) children reported lower respiratory symptoms with a mean age onset of 2.2 ± 2.5 years and only 63/69 (91%) performed specialist assessment at Respiratory Unit. Recurrent pneumonia (33%) and wheezing (31%) were the most reported symptoms. The first respiratory evaluation was performed after surgically correction of gastroesophageal reflux (GER) at mean age of 3.9 ± 4.2 years. Twenty nine patients have undergone to chest CT with contrast enhancement detecting localized atelectasis (41%), residual tracheal diverticulum (34%), bronchiectasis (31%), tracheal vascular compression (21%), tracheomalacia (17%) and esophageal diverticulum (14%). Fifty three patients have undergone to airways endoscopy detecting tracheomalacia (66%), residual tracheal diverticulum (26%), recurrent tracheoesophageal fistula (19%) and vocal cord paralysis (11%).

**Conclusions:**

Our study confirms that respiratory symptoms often complicate EA and TEF; their persistence despite medical and surgical treatment of GER means that other etiological hypothesis must be examined and that a complete respiratory diagnostic work up must be considered.

## Background

Children with congenital esophageal atresia (EA) and tracheoesophageal fistula (TEF) have chronic respiratory and digestive symptoms due to abnormal development of trachea and esophagus during intrauterine life. It is a rare condition that occurs in one per 3000 live birth [1]. Clinical presentation depends on the type of EA. Newborns with EA are not able to swallow saliva and milk and gaseous extension of the gastrointestinal tract combined with gastric reflux develops via the TEF. Infants with isolated TEF present with late onset symptoms as coughing and cyanosis during feeding, recurrent severe bronchitis and pneumonia.

The main goals of treatment are timely recognition of the underlying malformation, reduction of morbidity and provision of the best possible quality of life for patients and their parents.

Surgical treatment is the only therapeutic option and prognosis is good in 95 – 99% of children [2]. Preserving the original esophagus is the most important priority and is achieved in 90% of all cases by primary anastomosis with closure of the esophago-tracheal fistula. Even if the preferred approach is through a right-sided thoracotomy [3], in the past 10 years, thoracoscopic repair has become standard in specialized centers also for the correction of congenital and acquired TEF. A recent meta-analysis has confirmed the effectiveness of this approach, which seems to have outcomes not different from those of open surgery [4]. However, the difficulty of the technique, the worse acidosis and hypercapnia during the thoracoscopic repair [5], the lack of randomized controlled trials that evidence its efficacy, explain the skepticism toward this technique and its application only in few specialized centers.

Moreover, advances in surgical techniques and neonatal intensive care have resulted in improvement of survival and increase the prevalence of long term disease-related complications, including respiratory manifestations.

Chronic cough, recurrent bronchitis and pneumonia, wheezing and dyspnea are symptoms often reported by children or their parents resulting in decreased quality of life during infancy, adolescence and adult age [1, 6, 7]. Although respiratory morbidity tends to improve with age, lung impaired function could be detected also in asymptomatic children and adults [8] and long term lung damage – as bronchiectasis – may also develop as a consequence of repeated lower respiratory infections [9].

Recurrent esophageal stricture, esophageal dysmotility and gastroesophageal reflux may contribute worsening respiratory morbidity [10].

Tracheobronchomalacia is a frequent issue in this group of patients. Retrospective studies [11–15] report the incidence rate ranging from 24% [16] to 79% [9].

The presence of dysphagia in children with severe tracheomalacia suggests a relationship between gastrointestinal and pulmonary symptoms. In fact GER, dysmotility, anastomotic strictures are risk factors for aspiration and worsening tracheomalacia [17–19].

Even though this data are available for a limited cohort of children, DeBoer and al. have highlighted the need for a multidisciplinary evaluation that should be done more closely especially in the first years of life [9].

The purpose of our study is to describe the respiratory symptoms and the instrumental findings of a large group of children with EA and TEF referred at the Department of Pediatric Medicine of Bambino Gesù Children’s Hospital (Rome, Italy). Another aim of the study is to understand patients’ needs, improving and establishing a coordinated follow up still not defined by an international consensus.

## Methods

This is a retrospective study of 105 children affected by EA and TEF surgically repaired who referred to the Department of Pediatric Medicine of Bambino Gesù Children’s Hospital (Rome, Italy) for a multidisciplinary follow-up program after surgical correction between 2010 and 2015.

Demographic data, medical histories and results of diagnostic testing (chest computed tomography scan and flexible laringotracheobronchoscopy) were obtained from electronic medical record review with approval from the institutional review board.

The review of the instrumental tests performed at our institution has been made to detect residual anatomic or functional anomalies of airways and gastrointestinal tract that could explain the respiratory clinical pictures.

All patients underwent preoperative flexible laryngo-tracheobronchoscopy (LTBS) as part of a standardized preoperative diagnostic assessment to define the diagnosis, evaluate preoperative vocal cord motility and to cannulate the fistula when required and assess the presence of other associated anomalies. Repetition of flexible LTBS was performed when stridor, apnea, dysphonia or recurrent lower respiratory symptoms were present postoperatively or during the long-term follow-up period.

Multidetector computed thomography (MDCT) with contrast enhancement and during inspiration and expiration phase without general anesthesia was performed preoperatively to evaluate complex associated malformations (cardiac, vascular, tracheal, bronchial etc.) and when recurrent lower respiratory symptoms were reported during the postoperative and long-term follow-up period.

All newborns were preoperatively screened for major associated abnormalities. In particular the screening included: chest and abdominal X-ray searching for vertebral anomalies; cerebral, renal and abdominal ultrasound for associated abnormalities; electrocardiographic and echocardiographic

assessment to define out heart anatomy and function.

The thoracotomy approach was performed to repair EA with or without TEF.

Tracheostomy was considered when ventilation weaning was not achievable after 4 weeks overall. Upper respiratory symptoms were defined as cough associated to sore throat, runny nose, nasal congestion and sneezing without bronchial or pulmonary involvement.

Lower respiratory symptoms were defined as cough associated to bronchitis, pneumonia or wheezing.

Children without apnea, stridor and lower respiratory symptoms were excluded from respiratory assessment during the postoperative and the long-term follow-up period.

Definition of positive pH ± impedance study was made according NASPGHAN/ESPGHAN standard protocol: the exam has been defined positive when the reflux index (percentage of the total time that the esophageal pH is <4) was 11.7% in infants and 5.4% in children [20].

Tracheomalacia was defined as a reduction at least 50% of tracheal lumen with spontaneous quiet breathing detected during flexible bronchoscopy made under light sedation with inhalatory anesthesia with sevoflurane [21].

Tracheomalacia detected with inspiratory-expiratory MDCT with contrast enhancement was defined as dynamic tracheal collapse during end-expiration phase [22]. Due to neurodevelopmental sequelae and presence of tracheostomy, only 26 pre and post bronchodilator spirometry data were available for children aged over 6 years and followed at our Respiratory Unit. Spirometry was performed according to the American Thoracic Society/European Respiratory Society standards [23], using a Cosmed Quarck spirometer (Cosmed, Rome, Italy). The subjects were trained to perform spirometry and the best value of three measures was considered. The pulmonary function test was performed standing upright and wearing a noseclip. The results were expressed as a percentage of predicted values for height and sex.

The collected data are expressed as numbers, means ± SD and percentages. Data were analyzed using Student *t* test and χ^2^ analysis. Odds ratio (OR) with 95% confidence intervals (CI) were calculated from 2 × 2 contingency tables standard methods. A *p* value of less than 0.05 was considered to be statistically significant.

## Results

One hundred five Caucasian children with esophageal atresia and tracheoesophageal fistula followed at the Department of Pediatric Medicine of Bambino Gesù Children’s Hospital for a period of 6 years were included in the study.

The mean age at the time of enrollment was 7.4 ± 4.8 years. 57/105 patients (54%) were male. Mean gestational age was 36.9 ± 2.57 weeks (range 28 – 41 weeks) and mean neonatal weight was 2579.6 ± 609.8 g (range 760 – 3980 g). Data on mean gestational age and mean neonatal weight were unavailable respectively for 16 and 21 patients. 55/105 patients (52%) had associated malformations: heart disease and vascular anomalies were the most described defects and affected respectively 46/105 (43%) and 9/105 (9%) children. 20/105 children (19%) had syndromic picture and VACTERL association was diagnosed in 17/105 patients (16%). Malformations or syndromes were not found in 30/105 children (29%).

**Fig. 1:**
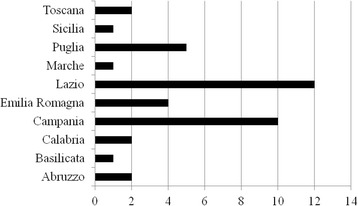
Patients coming from other Italian hospitals where they were surgically treated

Surgical repair of EA with TEF was performed at Neonatal Surgery Unit of our hospital in 64/105 patients (61%). The remaining patients were sent from other Italian institutes after surgical treatment (Fig. 1). Data on fistula type were available for 103 cases and type C fistula was the most frequent EA variety (86%), followed by B (8%), A (4%) and E (1%) types.

Demographic, medical history and clinical data are clearly illustrated in Table 1.

**Table 1:** Demographic and clinical features of study population

Total, n	105
Male, n (%)	57 (54%)
Mean gestational age, weeks ^a^	36.9 ± 2.55
Mean neonatal weight, gr ^a^	2579.6 ± 609
Mean age at enrollement, years	7.4 ± 4.8
Malformations, n (%)	55 (52%)
Cardiac anomalies	46 (44%)
Vascular anomalies	9 (9%)
Genitourinary anomalies	7 (7%)
Pulmonary anomalies	4 (4%)
Gastrointestinal anomalies	4 (4%)
Skeletal anomalies	2 (2%)
Syndromes, n (%)	20 (19%)
VACTERL association	17 (16%)
Down syndrome	1 (1%)
CHARGE syndrome	1 (1%)
Anophtalmia Esophageal Genital syndrome	1 (1%)
Types of fistula, n (%)	103 (98%)
C	90 (86%)
B	8 (8%)
A	4 (4%)
E	1 (1%)

^a^Data about mean gestational age and mean neonatal weight were available respectively for 89 and 84 patients

Minor leak of anastomosis occurring in the first month of life interested 12/105 (11%) children and was most frequent in those initially surgically treated at our centre (14%). In these patients oral milk intake was restricted and enteral or/and parenteral nutrition support was guaranteed.

Long term digestive complications occurred in 90/105 (86%) patients: 6/105 (6%) had esophagitis, 44/105 (42%) had esophageal stenosis and 72/105 (69%) had gastroesophageal reflux (GER).

Gastroesophageal reflux was detected with pH ± impedenziometry (76%), swallow contrast examination (21%), and gastric emptying scintigraphy (3%). It was more frequent in patients surgically treated at birth at other centers (78%). More details are showed in Table 2.

**Table 2:** Details of short and long term digestive complications occurred in study population

	Patients treated at our centre	Patients treated at other hospital	Total population’s study
Number	64	41	105
Leak anastomosis, n. (%)	9 (14%)	3 (7%)	12 (11%)
Esophagitis, n. (%)	4 (6%)	2 (5%)	6 (6%)
Esophageal stenosis, n. (%)	29 (45%)	15 (37%)	44 (42%)
Gastroesophagal reflux, n. (%)	40 (63%)	32 (78%)	72 (69%)

Children with esophagitis were treated medically and those with esophageal stricture (esophageal anastomosis ≤5 mm in diameter) underwent to repeated endoscopic balloon dilatation under general anaesthesia. All patients with GER were at first treated with protonic inhibitor pump but only 26/72 (36%) underwent to Nissen fundoplication at mean age of 2.6 ± 3 years (range 2 – 120 months) because they not resolved GER symptoms showing also failure to thrive. All patients requiring medical, ballon dilatation and/or surgical treatment showed improvement in their gastrointestinal symptoms. Only 19/105 (18%) patients had personal history of atopy defined by the combination of clinical symptoms, skin prick tests and specific IgE levels.

Of the entire sample, 82/105 (78%) children reported respiratory symptoms: lower respiratory symptoms occurred in 69/82 (84%) patients and upper respiratory symptoms in 13/82 (16%). The onset of respiratory problems was almost the same in the two group, respectively 2.2 ± 2.5 years and 2.2 ± 1.7 years. Only 63/69 (91%) patients with lower respiratory symptoms performed a pediatric respiratory assessment and 8 of them had tracheostomy for acute respiratory failure and prolonged intubation arose in the postoperative period; the remaining 6 (9%) children had aspiration pneumonia in the immediately postoperative period and a specialist evaluation wasn’t requested.

**Fig. 2:**
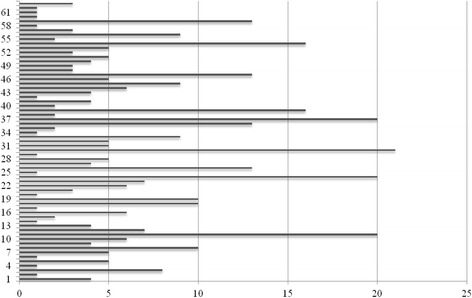
Number of respiratory evaluations made by 63 children with LRTI over the period of follow-up

The first respiratory evaluation was made at mean age of 3.9 ± 4.2 years. Recurrent pneumonia (33%) and wheezing (31%) were the most reported lower symptoms followed by stridor (3%) and apnea (2%). Based on their symptoms, patients performed repeated specialist evaluations over the period of follow-up (Fig. 2).

According to the clinical history of recurrent lower chest infections, 29 and 53 children underwent to chest CT with contrast enhancement and flexible bronchoscopy in order to study airways and their relationships with the vascular structures. CT scan was pathological in 28 patients and localized atelectasis (41%), tracheal diverticulum (34%), bronchiectasis (31%), tracheal vascular compression (21%), tracheomalacia (17%), esophageal diverticulum and bronchial stenosis (14%), recurrent tracheoesophageal fistula (7%) were the most detected findings. Flexible bronchoscopy performed under light sedation was pathological in 47 cases: tracheomalacia (66%), tracheal diverticulum (26%) and recurrent tracheoesophageal fistula (19%) were mostly showed.

Except for vocal cord paralysis, subglottic stenosis, bronchomalacia and the other airway complications were more frequent in children coming from other hospitals.

Twenty six children aged over 6 years were able to perform spirometry: restrictive ventilatory defect was the most detected (15/26), followed by normal (8/26), obstructive (2/26) and mixed ventilatory patterns (1/26).

Total instrumental findings and distribution of the main airway complications are illustrated in Tables 3 and 4.

**Table 3:** Instrumental findings detected in patients undergone to CT with contrast enhancement and flexible laringotracheobronchoscopy

Findings	LTBS, n (%)	CT scan, n (%)
	Total 53	Total 29
Tracheomalacia	35 (66%)	5 (17%)
Tracheal diverticulum	14 (26%)	10 (34%)
Lobar atelectasis	0	12 (41%)
Recurrent tracheoesophageal fistula	10 (19%)	2 (7%)
Bronchiectasis	0	9 (31%)
Tracheal vascular compression	3 (6%)	6 (21%)
Vocal cord paresis	6 (11%)	0
Bronchial stenosis	2 (4%)	4 (14%)
Esophageal diverticulum	0	4 (14%)
Subglottic stenosis	3 (6%)	2 (7%)
Pulmonary hypoplasia	0	3 (10%)
Larynx cleft	2 (4%)	0
Bronchomalacia	1 (2%)	1 (3%)
Coanal stenosis	1 (2%)	0
Laryngomalacia	1 (2%)	0
Vocal cord nodule	1 (2%)	0

**Table 4:** Detailed distribution of the main airway complications in children coming from our surgical unit and other surgical institutions

Airway complications	Patients treated at our centre	Patients treated at other hospital
Number	64	41
Tracheomalacia	19 (30%)	16 (39%)
Tracheal diverticulum	5 (8%)	11 (27%)
Recurrent tracheoesophageal fistula	3 (5%)	7 (17%)
Lobar atelectasis	2 (3%)	10 (24%)
Bronchiectasis	3 (5%)	6 (14%)
Bronchomalacia	2 (3%)	0
Vocal cord paresis	5 (8%)	1 (2%)
Subglottic stenosis	2 (3%)	0
Larynx cleft	1 (2%)	1 (2%)

Tracheostomy was initially required for four children with bilateral vocal cord paralysis; the remaining two patients underwent to CO2 laser-posterior cordotomy but only in one case tracheostomy was performed. Two cases of laryngeal cleft were corrected using endoscopic closure.

The injection of fibrin glue into the submucosa of the lateral walls of the fistula was used in three patients but only one case was successful. Laser therapy was used in one patient with benefit. The re-thoracotomy and the re-ligation were performed in all the other cases of recurrent TEF.

Tracheal diverticulum and tracheomalacia were conservatively treated and techniques of airway clearance were used to prevent respiratory exacerbations. Only one patient with residual bronchial stenosis underwent to bronchoplasty. Parents of patients receiving the above treatments reported improvement of “barking cough” and reduction of severe respiratory exacerbations requiring antibiotic therapy also performed in a hospital setting.

Comparing patients with and without lower respiratory symptoms, there was a trend associating the lower respiratory symptoms with male gender (OR 1.8300), type C fistula (OR 1.3000), VACTERL association (OR 1.8600), leak of anastomosis (OR 1.0500) and GER (OR 1.3800) but it was not statistically significant. As expected, atopy was significantly associated with lower respiratory disturbances (OR 12.350, *p* 0.0032; 95% CI 1.5758 – 96.8387). All statistical evaluations are showed in Table 5.

**Table 5:** Variables associated with respiratory tract symptoms: comparison between children with and without low respiratory symptoms

Variables	Odds ratio, *p* value
Male	1.8300, *p* = 0.1437
C type fistula^b^	1.3000, *p* = 0.6145
Malformations	0.3400, *p* = 0.0114
Heart disease	0.3400, *p* = 0.0099
VACTERL association	1.8600, *p* = 0.3074
Leak of anastomosis	1.0500, *p* = 0.9411
Long term digestive complications^a^	0.9500, *p* = 0.9331
Gastroesophageal reflux	1.3800, *p* = 0.4553
Atopy	12.350, *p* = 0.0032

^a^gastroesophageal reflux, esophagitis and esophageal stenosis

^b^Data on fistula type were available for 103 cases

In addition, the mean age onset of respiratory complaints was 1.3 ± 1.25 years in patients with associated heart disease: the onset was precocious if compared with children without cardiac anomalies (mean age onset 2.7 ± 2.8 years) and the difference was weakly statistically significant (*p* 0.0521; 95% CI 3.2853 – 32.4187).

## Discussion

Respiratory morbidity is still highly prevalent in EA and TEF survivors despite the improvement of perinatal and surgical care, the greater awareness and more aggressive therapeutic strategies concerning associated morbidity. We must consider that many factors contributing to the clinical complexity of patients play a pivotal role for the respiratory morbidity of EA and TEF patients.

Firstly, the associated congenital malformations frequently described in male patients [24]. Heart abnormalities and VACTERL association are equally distributed in our sample between male and female patients; they are mostly described in our study population if compared with data of the international literature, in which the rate of malformations and defects of VACTERL association is respectively about 31.6% and 9.6% and heart abnormalities interest about 29.4% of children [2]. Considered alone, isolated malformations, abnormalities related to VACTERL association and heart defects are not significantly related to respiratory complaints. This observation is consistent with that reported by Legrand and coworkers [25].

Secondly, airway anomalies such as tracheomalacia that is reported in more than half of our sample, tracheobronchial malformations and lung hypoplasia [6, 9] contribute to recurrent respiratory exacerbations through the impairment of the mucociliary transport.

In addition, esophageal dismotility due to esophageal stenosis and GER may cause and/or worsen respiratory function. Even if the relationship between gastrointestinal disturbances and respiratory symptoms is not always clear [25], in our patients the rate of esophageal strictures and GER is in line with that reported by Allin [26] and Acher [27].

Although several articles on the main long term respiratory complications in EA children have been published [1, 6, 8, 9, 12, 25, 28–31], a shared management design has never been developed. Only a recent paper has proposed a simplified management algorithm of pulmonary complications resulting from the review of 26 original articles that exclusively investigated respiratory disease in EA survivors [32].

In order to understand better the need of this group of children and improve their care, we have described a large case series that reinforces some of what other smaller studies have shown about respiratory symptoms and bronchoscopic findings [9].

We want to point out that the present study is a retrospective study carried out in a real life situation, so we decided the diagnostic approach in reason of clinical symptoms reported by patients and their parents. Consistent with the international literature [10], the EA with distal TEF (Gross type C) was the most detected in our sample.

Data about the rate of leak of anastomosis in EA patients are not available in scientific literature. In our sample this short term digestive complication is present in patients treated at our institution twice more than children initially treated in other hospitals. This observation is probably an overestimation due to the poor received information about the post-operative complications interesting the second group of children. Anyway, the described minor leak of anastomosis – that was always conservatively treated – appears not related to respiratory symptoms.

Limited to the long term digestive complications, the rate of esophageal stricture and GER is consistent with that reported by other studies describing these complications in 18% to 60% of patients with EA [25, 33–35].

Children with symptoms of nausea, vomiting, dysphagia or failure to thrive were examined in order to exclude GER that was detected with pH ± impedance in the major number of cases. According to recent ESPGHAN-NASPGHAN Guidelines for the Evaluation and Treatment of Gastrointestinal and Nutritional Complications in Children With Esophageal Atresia-Tracheoesophageal Fistula, all children (including asymptomatic patients) should undergo monitoring of GER through impedance/pH-metry and/or endoscopy at time of discontinuation of anti-acid treatment and during long-term follow-up [36]. Even if esophagogastroduodenoscopy with biopsy remains the primary surveillance tool of GER disease, less-invasive modalities can also be evaluated. In particular, the multichannel intraluminal impedance and pH monitoring appear to correlate strongly with esophageal histology and may provide sufficient information to guide treatment in patients with GER [37]. In our sample, the rate of Nissen fundoplication treatment is greater in children initially surgically treated at other hospitals. This observation is probably influenced by the selection bias represented by the complexity of this group of children that was referred at our institution just because of the greater clinical complexity developed in the postoperative period.

About the rate of atopy, it is lower in our paper than usually reported in scientific literature [38] and, as expected, it seems to be independently associated with higher risk of lower respiratory disturbances as also previously found by Sistonen et al. [38].

Even if symptoms occur in the first 3 years of life, their onset is precocious in children with heart disease. It is likely that cardiac disease [39, 40] and atopy [41] promote the onset of symptoms through the reduction of the threshold of the bronchial reactivity that is intrinsically linked to the congenital malformative picture, thus contributing to the respiratory morbidity.

Except for cases with aspiration pneumonia in the immediately post-operative period, the most frequent respiratory symptoms occurring in the first 3 years of life are recurrent pneumonia and wheezing, the rate of which is in line with that reported by other authors [9].

In our study, the delay in referral of children with respiratory symptoms to pneumologist is particularly interesting.

The delay is probably due to the attribution of respiratory complaints to gastroesophageal reflux for which some patients performed medical and then surgical therapy. Only after the verification of failure of the above therapeutic options, children were sent to a pediatric respiratory specialist.

The described tracheomalacia, residual tracheal diverticulum and recurrent TEF have surely contributed to the lower respiratory exacerbations as reported by other studies [42–44].

The rate of detected tracheomalacia is in line with that described in scientific literature [9, 16]. Because of only few case series describe the presence of residual tracheal diverticulum in children underwent to correction of EA and TEF [42, 43], it’s not possible to estimate the real frequency of this complication. Only the frequency of recurrent TEF in our study population is higher than previously described [44] and also this finding is greater in children surgically corrected at other centers: this observation is probably influenced by the same selection bias previously discussed for children undergoing Nissen fundoplicatio.

In our study population, chest CT scan with contrast enhancement is useful in the search of vascular anomalies, tracheal compression or esophageal diverticulum not otherwise detectable with flexible bronchoscopy. Furthermore, it is able to show atelectasis that we interpreted as the effect of disventilation due to mucus plugging and the pulmonary damage due to recurrent respiratory exacerbations. In the latter case, the greater frequency of bronchiectasis is most likely influenced by the higher mean age of the enrolled patients that could justify the establishment of lung damage. Abnormal pulmonary function is well described in patients with EA and TEF and the restrictive ventilatory impairment prevails over the obstructive and the mixed ones [30]. Also in our sample we observed a higher frequency of restrictive defects (58%) with obstructive, mixed and normal ventilatory pattern respectively in 8%, 4% and 31% of patients. Restriction may result from surgical trauma, repeated aspiration, or recurrent chest infections, as well as from associated thoracic musculoskeletal defects such as postoperative rib fusions, scoliosis or other chest deformities.

In interpreting the provided data, the limitations of retrospective analysis must be considered.

The limitations include primarily patients selection bias: examined patients were surgically treated not only at our institution and children coming from other centers often showed a greater clinical complexity due to postoperative complications.

Moreover, because not all participants had bronchoscopy, thorax CT, impedance testing and spirometry we cannot be certain that those not tested had normal findings, so our estimated prevalence of abnormality is likely underestimated.

Nevertheless, even though we are aware of the difficult to generalize the proposed data because describing the experience of a single center, our study confirms that respiratory symptoms are frequent in a large group of pediatric patients with EA and TEF and mostly develop in the first 3 years of life and that residual anatomical and dysfunctional airway anomalies contribute to their presentation. As showed by Fig. 2 describing the rate of medical evaluation in the study period, the respiratory burden among children with EA and TEF is high, so the need of strict respiratory assessment in symptomatic children becomes imperative.

In our opinion, our findings add weight to the need of routine surveillance protocols and close monitoring of these patients from the neonatal period onwards.

To date, although the need for careful multidisciplinary follow-up is highlighted, no recommendations on the respiratory management of infants and children with EA and TEF are available. The only available algorithm [32] suggests the stratification of disease severity on the basis of recurrence of respiratory symptoms and functional and instrumental abnormalities, providing a regular tertiary care follow up only for patients having moderate to severe airway disease.

Conversely, on the basis of our direct experience and the results provided by our study, we believe that all children, after the first 12 months of life, should be monitored at least every 6 months up to 36 months in a multidisciplinary pediatric setting for the evaluation of clinical history, growth and relevant symptoms.

Other professional figures, such as gastroenterologist, dietitian, cardiologist, otorhinolaryngologist, neuropsychiatrist, orthopedist and physioterapist could be variously involved in relation to the presence of gastrointestinal symptoms, poor growth, heart or airway abnormalities, neuromotor delay, congenital or acquired skeletal malformations. Respiratory evaluation should be performed when recurrent pneumonia (>2 episode/years), recurrent bronchitis or unexplained wheezing, persistent wet cough, nocturnal apnea and exercise intolerance occur. Also children with abnormalities on thorax X-ray, moderate to severe tracheomalacia and tracheostomy should be evaluated by a pulmonologist. The respiratory assessment should provide for chest X-ray at the first visit in order to define the lung baseline condition and whenever there is a suspicion of pneumonia. Nocturnal pulse oximetry and forced oscillation technique (FOT) could be performed once a year. The first one would detect desaturations deserving of other insights to exclude heart problems, central or obstructive respiratory events. Because it is provided that FOT yields concordant information of lung function with other conventional methods [45], this technique could be used for monitoring pulmonary function in the pre-school children.The purpose of the above suggested clinical assessment is to manage respiratory symptoms through medical treatment and chest physiotherapy, prevent lower respiratory exacerbation with precocious antibiotic therapy and gather information enabling the clinician to suspect an underlyng condition responsible of persistence of symptoms and requiring additional investigations such us bronchoscopy and CT scan. Because of the detection of associated vascular abnormalities determining airway compression in 21% of patients, we believe that thorax CT scan study should be completed with angiography.

Children with more than one radiographically confirmed episode of lobar pneumonia, chronic wet cough and suspected bronchiectasis must also be evaluated for aspiration with assessment for lipid laden macrophages on bronchoalveolar lavage (BAL). The last one should also be useful to rule out colonization of the airway by bacterial and other respiratory pathogens. The suggested follow-up program could avoid the underestimation of respiratory morbidity in this group of patients allowing the precocious and better management of the respiratory long term complications.

## Conclusions

Despite respiratory symptoms and abnormal findings on flexible bronchoscopy and CT evaluation, children’s symptoms are often attributed to reflux and esophageal abnormalities. Symptoms may be worsened by gastroesophageal reflux, but it can’t be considered the main etiological factor. Residual tracheomalacia, tracheal pouch or recurrent TEF can justify the recurrence of respiratory symptoms: the detection of these underling conditions is useful for the choice of the best surgical or medical treatment that could prevent respiratory exacerbations and long term dysfunctions. So, close monitoring of patients must be carry out at least in the first 3 years of life, giving special attention to children with cardiac disease and atopy state for which exalted bronchial reactivity can activate and maintain a “vicious circle” that further affects respiratory outcomes. As a result, because children with EA and TEF have interrelated diagnoses from multiple systems, a multidisciplinary team including a pulmonologist becomes imperative to improve the management and harmonize the care of this group of patients.

## Abbreviations

BAL: Bronchoalveolar lavage
CHARGE: Coloboma, heart defect, atresia choanae, retarded growth and development, genital abnormality and ear abnormality
CI: Confidence interval
EA: Esophageal atresia
FOT: Forced oscillation technique
GER: Gastroesophageal reflux
LTBS: Laryngo-tracheobronchoscopy
MDCT`: Multidetector computed thomography
OR: Odd ratio
TEF: Tracheoesophageal fistula
VACTERL: Vertebral defects, anal atresia, cardiac defects, tracheo-esophageal fistula, renal anomalies, and limb abnormalities
